# The Impact of SARS-CoV-2 Infection on Premature Birth—Our Experience as COVID Center

**DOI:** 10.3390/medicina58050587

**Published:** 2022-04-25

**Authors:** Tina-Ioana Bobei, Bashar Haj Hamoud, Romina-Marina Sima, Gabriel-Petre Gorecki, Mircea-Octavian Poenaru, Octavian-Gabriel Olaru, Liana Ples

**Affiliations:** 1 Department PhD, IOSUD, “Carol Davila” University of Medicine and Pharmacy, 020021 Bucharest, Romania; tina-ioana.bobei@drd.umfcd.ro; 2 “Bucur” Maternity, Saint John Hospital, 012361 Bucharest, Romania; gabriel.gorecki@doc.utm.ro (G.-P.G.); mircea.poenaru@umfcd.ro (M.-O.P.); octavian_olaru@umfcd.ro (O.-G.O.); liana.ples@umfcd.ro (L.P.); 3 Department for Gynaecology, Obstetrics and Reproductive Medicine, Saarland University Hospital, Kirrberger Straße 100, Building 9, 66421 Homburg, Germany; bashar.hajhamoud@uks.eu; 4 Department of Obstetrics and Gynecology, Carol Davila University of Medicine and Pharmacy, 050474 Bucharest, Romania; 5 Faculty of Medicine, Titu Maiorescu University, 040441 Bucharest, Romania

**Keywords:** COVID-19, pregnant women, gestation age, preterm birth, birth rate, SARS-CoV-2

## Abstract

Information about the impact of SARS-CoV-2 infection on pregnant women is still limited and raises challenges, even as publications are increasing rapidly. The aim of the present study was to determine the impact of SARS-CoV-2 infection on preterm birth pregnancies. We performed a prospective, observational study in a COVID-only hospital, which included 34 pregnant women with SARS-CoV-2 infection and preterm birth compared with a control group of 48 healthy women with preterm birth. The rate of cesarean delivery was 82% in the study group versus 6% for the control group. We observed a strong correlation between premature birth and the presence of COVID-19 symptoms (cough *p* = 0.029, fever *p* = 0.001, and chills *p* = 0.001). The risk for premature birth is correlated to a lower value of oxygen saturation (*p* = 0.001) and extensive radiologic pulmonary lesions (*p* = 0.025). The COVID-19 pregnant women with preterm delivery were older, and experienced an exacerbation of severe respiratory symptoms, decreased saturation of oxygen, increased inflammatory markers, severe pulmonary lesions and decreased lymphocytes.

## 1. Introduction

Coronavirus disease-2019 (COVID-19) was declared a pandemic on 11th March 2020 [1]. For pregnant women, the mortality rate in cases of SARS-CoV-2 infection was reported at around 25% in the early studies [2]. These studies found no evidence that indicates an increased susceptibility in pregnant women. However, more recent studies suggest a role of this virus in mortality and morbidity due to cardiorespiratory and immune system involvement, which may determine an abnormal response to SARS-CoV-2 infection in pregnancy [3,4,5].

Information about SARS-CoV-2 infection in pregnant women, the fetus, and the neonatal prognosis is still limited even if the number of published articles is increasing. There are studies that describe the prevalence of maternal and neonatal SARS-CoV-2 outcomes [1,2] and suggest a possible increased risk of preterm delivery in cases of pregnant women with COVID-19 [3]. The aim of the present study was to evaluate the impact of SARS-CoV-2 infection on preterm birth among COVID-positive pregnant women.

## 2. Methods

We conducted a prospective cohort observational study (based on STROBE Statement) which included pregnant women with SARS-CoV-2 infection and preterm delivery. Patients delivered at “Saint John” Hospital, “Bucur” Maternity, a tertiary COVID-only health facility. Since 19 March 2020, “Bucur” Maternity was destinated by the Romanian Ministry of Health as the Department of Obstetrics and Gynecology responsible only for obstetrical and gynecological SARS-CoV-2 infection pathology associated. 

The center where the study was conducted had an operating permit from the Bucharest Public Health Directorate for only 17 places in the obstetrics and gynecology ward and six places in the intensive care unit exclusive for COVID-19 patients. In this situation, only pregnant SARS-CoV-2-positive patients were admitted and cared for and we acted as a tertiary referral center for the whole south part of Romania (more than half of the Romanian population which is about 19 million persons).

Therefore, among the whole cohort of patients with SARS-CoV-2 infection, we selected women with preterm delivery from March 2020 to June 2021, which constituted the study group. The study obtained the approval of the ethical committee. Patients were included after signing their informed consent. We compared the study group with SARS-CoV-2 infection and preterm birth (COVID-19) with a historical control group of healthy women with preterm birth (non-COVID-19) who were hospitalized between March 2018 and March 2020 in the same department. The study was non-randomized. The control group was selected using the DRG (Diagnosis-Related-Group) code O.60 for preterm delivery.

The inclusion criteria were: singleton pregnancy, spontaneous pregnancy, gestational age between 24 and 36 weeks, RT-PCR (Real Time-Polymerase Chain Reaction) test positive for SARS-CoV-2, live fetus (ultrasound fetal viability), and adult women (range: 18–47).

The exclusion criteria were: age under 18 years, ART (Assisted Reproduction Techniques), pregnancy-associated pathologies, stillbirth, multiple pregnancies, previous premature birth, cervical incompetence, presence of pessary or cerclage, and the refusal of different investigations or treatment. 

The analyzed parameters were: maternal age, obstetrical history of abortion, parity, rupture of membranes, gestational age at birth, days of ongoing COVID infection, birth type, COVID-19 symptoms, maternal and newborn evolution, CRP (C-Reactive Protein) level, leucocyte and lymphocyte values, and thoracic X-ray. All variables were obtained from the patient’s observation file.

Statistical analysis was performed using Statistical Package for Social Sciences SPSS Statistic software, version 23 (Armonk, New York, NY, USA). (SPSS). Frequencies and descriptive data as well as correlations were recorded. A *p*-value < 0.05 was considered statistically significant. Pearson correlation was used for bivariate variables.

## 3. Results

In the period of study, 377 pregnancies with COVID 19 were admitted and 238 births (204 at term and 34 preterm births) occurred in our tertiary center. The overall rate of premature births was 14.28%; this rate was 8.2% during the previous non-COVID period in our clinic.

Our study included 34 pregnant patients with SARS-CoV-2 infection and preterm delivery compared with 48 patients from the control group.

Within the control group, we had eight cases of extremely preterm birth (<28 weeks gestational age, seven cases of moderately preterm birth (28 weeks to 31 weeks gestational age), and 33 cases of late preterm birth (32 weeks to 36 weeks+ 6 days gestational age), whereas within the SARS-CoV-2 group, there were three cases of extremely preterm births, four cases of moderate preterm, and 27 cases of late preterm births. The compared control and study group characteristics are illustrated in Table 1.

The obstetrical characteristics of the studied groups were similar. There was a higher percentage of pregnant women over 41 years old with COVID-19 who gave birth prematurely compared with healthy women. Within the COVID-19 group, we observed a lower percentage of extremely preterm births and higher percentages of moderate to late preterm births compared with the control. The major and significant difference among the two studied groups was the highest rate of cesarean section performed in COVID-19 patients for preterm delivery. The rate of cesarean delivery was increased in the COVID-19 period due to clinical indications, severe maternal clinical conditions and fetal distress (Table 2), which also justifies the increased rate of induced preterm births.

We observed that the rate of cesarean section in the COVID-19 group of preterm labor was increased (82%) in this period and that two of the most frequent indications were the symptoms exacerbation, such as severe acute respiratory distress (33.5%) and fetal distress (24.6%), in comparison with only 6% in the non-COVID-19 group with previous C-section and fetal distress as indications.

The mean gestational age for COVID-19 diagnosis was 33.8 ± 8.2 weeks, with 7.4% of women being diagnosed in the first trimester, 9.7% in the second trimester, and 82.9% in the third trimester of pregnancy. Patients were asymptomatic in 63.2% of cases. The most common symptoms were cough (23%), followed by shortness of breath (21.6%), and fever (12.2%). Overall, 82% had cesarean section and 18% had a vaginal birth.

The distribution of the group according to the interval since the positive result of the RT-PCR test is illustrated in Table 3, from which we can observe that the highest proportion of premature births is represented by patients in the first four days of disease.

Regarding the biological markers, we found an inversed correlation between the value of CRP (C-Reactive Protein) and gestational age at birth (*p* = 0.001) or leucocyte count (*p* = 0.001). So, the risk of premature birth increases with the augmented value of inflammatory markers (Table 4). The lymphocyte count was indirectly correlated with the COVID-19 symptoms and the necessity of delivery. Patients with lower number of lymphocytes had more severe COVID-19 symptoms and more often underwent induced preterm delivery (*p* = 0.001).

In the group of pregnant women with SARS-CoV-2 infection, a strong correlation was established between premature birth and the presence of COVID-19 symptoms (cough *p* = 0.029, fever *p* = *0*.001, and chills *p* = 0.001). The risk for premature birth is correlated with the decreased value for oxygen saturation (*p* = 0.001) and extensive radiologic pulmonary lesions (*p* = 0.025); the chest X-ray were taken with all standard protective measures for the patient. So, the risk for premature birth increased with moderate and severe infection with SARS-CoV-2 in the present study.

We also identified a strong correlation between severe maternal symptoms such as respiratory distress and newborns’ outcome (*p* = 0.001). Women with severe respiratory distress delivered newborns who required neonatal intensive care. In the study group, the rate of perinatal death was about 5.9%, mainly related to prematurity. Gestational age at diagnosis, birth weight, and maternal ventilatory support were the main risk factors associated with adverse fetal outcomes.

The severity of symptomatology was correlated with cesarean birth rate (*p* = 0.034). Patients who experienced an exacerbation of severe respiratory symptoms delivered mostly by cesarean section. A total of 20.59% of patients had an unfavorable outcome, with mechanical ventilation and intubation before or after delivery and the transfer in intensive care units that could provide a higher degree of support.

## 4. Discussions

Pregnant women have a higher susceptibility to respiratory pathogens due to the adaptive anatomical and physiological changes in the respiratory system that occur during pregnancy, and these viral infections can induce pregnancy complications [3,6].

The purpose of this study was to determine the impact of SARS-CoV-2 infection on preterm birth, and we demonstrated that the rate of cesarean delivery was higher in the COVID-19 period due to severe maternal clinical conditions and fetal distress in comparison with the non-COVID-19 group. A strong correlation was observed between severe maternal symptoms such as respiratory distress and poor newborns’ outcome (*p* = 0.001).

Oltean et al.’s review suggests elevated rates of ICU admission, C-sections, pre-eclampsia, placenta praevia, gestational diabetes, placental abruption, preterm birth, and elevated levels of CRP in women with COVID-19 in comparison to pregnant women without SARS-CoV-2 [7]. In our study, we identified that an increased CRP level is a risk factor for preterm birth.

Women with severe symptoms are more susceptible to adverse outcomes such as premature birth, even if pregnancy itself does not appear to aggravate the course of clinical characteristics or symptomatology of COVID-19 pneumonia [3,8]. Our study revealed the same pattern.

Increased levels of inflammatory markers are a risk factor that associated with other immunologically mediated processes are thought to play a role in the preterm birth syndrome [9].

According to Silva et al., the inflammatory status can promote conditions such as pre-eclampsia, intrauterine growth restriction, or premature birth [10]. Based on our clinical research, the gestational age decreased with the increased levels of inflammatory markers, so the inflammatory status may be the promotor factor for preterm delivery also in SARS-CoV-2-associated pregnancies.

We found a decreased rate of extremely preterm births in the COVID-19 group and an increased rate of moderate to late preterm births within the control group. In a Danish review, the rate of extremely premature birth also decreased during the COVID-19 lockdown [11].

As DiMascio et al. found, the main determinants of adverse perinatal outcomes in fetuses from mothers with COVID-19 infection are maternal ventilatory support, early gestational age at infection, and low birth weight [12]. We identified a strong correlation between severe maternal symptoms such as respiratory distress and newborns’ outcome. The results of our study showed that in pregnancies with COVID-19, the rate of perinatal death was also increased, mainly related to prematurity. Gestational age at diagnosis, birth weight, and maternal ventilatory support were the factors associated with adverse fetal outcomes.

Since the onset of the COVID-19 epidemic, pregnant women with suspected or confirmed SARS-CoV-2 infections have undergone cesarean delivery in the absence of other obstetric indications in order to reduce the risk of intrapartum transmission, as some authors showed [13]. According to Bellos’s meta-analysis, from currently available case series, a higher than expected number of preterm deliveries and cesarean sections are found in SARS-CoV-2 pregnancies [14]. Several studies have established that the incidence of premature and cesarean births increased during the pandemic period. There are cohort studies describing a limitation of risk in patients with severe forms of the disease, possibly with associated comorbidities. The main factors that may increase the risk of preterm labor, premature rupture of membranes, preterm birth, and abnormal fetal heart rate are fever and hypoxemia, but preterm births also occur in pregnant women with mild to moderate forms of the disease. Changes in prenatal care and increased stress during the pandemic may also increase the incidence of preterm births. One of the limitations of multiple studies is that spontaneous preterm birth is not differentiated from iatrogenic preterm birth. It appears that most cases of COVID-19 in the third trimester are delivered by planned cesarean section for the management of severe maternal illness, which may be partly explained by the obstetric decision to deliver due to the severity of the maternal infection (bilateral pneumonia with respiratory failure and shock) [14].

As can be seen in our results, the rate of preterm births and cesarean sections are increased due to severe maternal clinical conditions, and fetal distress compared to non-COVID patients. Unless fetal extraction is required to improve maternal oxygenation, SARS-CoV-2 infection is not an indication for delivery. There are several studies describing a direct association between preterm birth and COVID-19 infection [15,16]. From the onset of the pandemic, the majority of pregnant women confirmed with SARS-CoV-2 infection have given birth by cesarean section, with no other obstetrical indication [17].

Other poor outcomes in pregnant women with COVID-19, such as pre-eclampsia, preterm birth, and delivery by emergency cesarean section, are responsible in a cohort study for a high rate of fetal deaths [18]. In our study, there is a strong correlation between the type of delivery and COVID-19 symptoms. All symptomatic pregnant women delivered by cesarean section. In Allotey’s analysis, an increased rate of preterm birth was observed in the COVID-19 pandemic compared to the worldwide reference rate. The same finding was observed in our study as well. The vaginal preterm birth rate was relatively low (5–6%), which is comparable to the general population [19,20] as the rate in the present.

The placental characteristics of pregnant women confirmed with SARS-CoV-2 infection were associated with perinatal outcomes, and it was determined that placental patterns indicate no clear evidence of transplacental transmission of SARS-CoV-2 or significant impact on perinatal outcomes in both mild and severe cases [21]. Multiple studies have reported no evidence of SARS-CoV-2 presence in the placenta, amniotic fluid, or cord blood [15,22]. Since there is no proof of vertical transmission, COVID-19 is not an indication for cesarean section in order to prevent transmission during expulsion [15,23]. In our study, no newborn was confirmed with SARS-CoV-2 infection by RT-PCR testing at 24 h and 48 h after birth within.

Similar to the results of Papanou’s meta-analysis, the present study finds that the rate of preterm births increased during the pandemic due to iatrogenic involvement [24]. At the time when our study started, the vaccination of pregnant women against SARS-CoV-2 was not yet approved. As a consequence, no pregnant women were vaccinated and we could not determine what impact it would have had in reducing the risk of maternal, birth, and neonatal outcomes. According to Heather S. Lipkind et al., COVID-19 vaccination during pregnancy was not related to preterm birth or low gestational age at birth overall, compared with unvaccinated pregnant women [25]. The available data suggest that vaccination during pregnancy is associated with antibodies against SARS-CoV-2 transmission to the fetus, but the level of protection of the newborn provided by transplacental and breast milk antibodies is unclear [26].

The limitations of our study were: the reduced number of patient, the study was performed during the first period of the pandemic and there were no evidence about the vaccine anti SARS-CoV-2 on pregnant women. Meanwhile, the number of patient treated in our COVID exclusive center provided the largest cohort of the pregnancies infected with SARS-CoV-2 in our country and can be considered also the main strength.

The impact of the SARS-CoV-2 pandemic on obstetrical population and neonates is a subject of intense debate with the unpredictable outcome and frequent changes in pathogenicity and contagiosity of the virus variants. Considering morbidity and mortality of the prematurity SARS-CoV2 impact on premature birth is a matter of intense debate.

## 5. Conclusions

The present study investigates the main characteristics of COVID-19-associated preterm deliveries in a tertiary center providing exclusive care for those patients and having the highest level of experience in Romania. The pregnant women with COVID-19 with preterm delivery were older women, who experienced an exacerbation of severe respiratory symptoms, decreased saturation of oxygen, increased inflammatory markers, extensive pulmonary lesions and decreased lymphocytes. The preterm delivery was mainly induced and by cesarean section and the newborns required intensive care more often than the ones delivered by healthy mothers.

## Figures and Tables

**Table 1:** Clinical characteristics of the patients.

MATERNAL CHARACTERISTICS	COVID-19 PATIENTS (%)	NON-COVID-19 PATIENTS (%)	*p* VALUE
**MATERNAL AGE (YEARS)**			
**18–30**	41	52.1	<0.001
**31–40**	46.2	43.8	<0.001
**≥41**	12.5	4.2	<0.001
**GESTATIONAL AGE (WEEKS)**			
**<28**	7.7	16.7	<0.01
**28–31**	12.8	14.6	<0.01
**32–36**	79.5	68.8	<0.01
**BIRTH TYPE**			
**VAGINAL**	18	94	<0.001
**CESAREAN SECTION**	82	6	<0.001
**SPONTANOUS PRETERM BIRTHS**	24	84	0.024
**INDUCED BIRTH PRETERM BIRTHS**	76	16	0.012

**Table 2:** Cesarean section indications.

Cesarean Indications (Percentage)	COVID-19 Patients (*n* = 34)	Non-COVID-19 Patients (*n* = 48)
Previous C-section	4.7	67
Exacerbation of symptoms	33.5	-
Labor dystocia	37.2	-
Fetal distress	24.6	33

**Table 3:** COVID-19-related characteristics in the study group.

COVID-19 Characteristics	Days	Percentage (%)
Days of infection	1–4 days	59
	5–9 days	32
	10–14 days	9
Symptoms	asymptomatic	53
	mild	25.7
	moderate	11
	severe	10.3
Maternal outcome	curred	58.82
	improved	20.59
	transfer	20.59
Newborn outcome	good	41.2
	improved	50
	transfer	2.9
	dead	5.9

**Table 4:** CRP level.

CRP Level (Values Interval/mg/dL-Percentage)	COVID-19 Patients (*n* = 34)	Non-COVID-19 Patients (*n* = 48)
<0.5	7.8	52.1
0.5–49	73.4	43.7
50–99	13.6	2.1
>100	5.2	2.1
